# How do political coups disrupt Fiji's tourism? Impact assessment on ecotourism at Koroyanitu National Heritage Park (KNHP), Abaca

**DOI:** 10.1016/j.heliyon.2021.e07101

**Published:** 2021-05-28

**Authors:** Sakul Kundra, Mumtaz Alam, Mohammad Afsar Alam

**Affiliations:** Department of Social Science, College of Humanities and Education, Fiji National University, Natabua, Lautoka, PB 5529, Fiji

**Keywords:** Coups, Democratic government, Ecotourism, Fijian nationalism, Political impact, Post-coup recovery, KNHP, Fiji

## Abstract

The nexus between mass tourism and politics has been widely validated in tourism literature; nonetheless, the impacts of political putsches on ecotourism are understudied in the context of the Pacific Islands, i.e., Fiji. This study aims to investigate how Fiji's political upheavals impacted ecotourism after examining ecotourist arrivals and revenue generated at KNHP from its inception till 2018. Additionally, it presents a comparative analysis of Fiji coups on tourism and ecotourism and examines the recovery pattern of ecotourism in the post-coup stage. The study implies both empirical and non-empirical methods. This research is based on field visits to the Abaca ecotourism project, Lautoka, from 2017 to 2019, and has employed quantitative data of Abaca's tourist revenue records; supplemented by conducting oral unstructured interviews of the Abaca project manager through *talanoa* sessions. The collected data were analyzed using Microsoft Excel 2010 and STATA and Augmented Dickey Fullar (ADF) test to check the stationarity. The research has defied the antecedent arguments of the failure of ecotourism projects in Fiji and made a first-ever comparative analysis of ecotourism data with mass tourism in relation to post-coup recovery tenure. It postulates an apparent correlation of the success of this ecotourism park with the democratically elected government of Fiji. The Abaca ecotourist park displayed potential to emerge as an ecotourism hub in the entire South Pacific, provided the democratically elected government could sustain in Fiji, which has been tagged as ‘coup-coup land’. Henceforth, this study can be replicated for similar destinations in the world.

## 1 Introduction

Tourism is closely related to political action, which is also used as a tool for political and economic change; for example, where calls are made for tourist boycotts of countries with undesirable political regimes or where tourism is used to initiate political discussions on fair trade (Butler and Suntikul 2017). There has been increased tourism literature on the politics of tourism (Hall, 1994; Dredge et al., 2011) and on political instability and its consequence on tourism (Richter 1999). It has been more generally noted that tourism requires security and stability at its location, where there should be little or no threat to personal safety and minimal commercial risk, which is not the case for violent ‘totalitarian’ states (Hall and Ringer 2000). Political change has affected the patterns, processes and directions of tourism development (Hall and Jenkins 2010) and Fiji Islands' tourism is not an exception, where it had to suffer four political upheavals that had a considerable impact on its tourism sector.

Fiji's image as a tourist destination has changed from that of Cannibal Isles to Friendly Fijians—an image that has to bear the shocks of political instability. It has been especially tarnished by the four political putsches the country witnessed in May and September 1987, 2000 and 2006. It seemed to threaten potential tourists' physical safety and disturbed their holiday experience. Gradually, as Fiji suffered adverse impacts in its mass tourism, there was a move towards a sustainable form of tourism i.e., ecotourism. But tourist inflow, revenue income and the recovery period after political upheavals worked against development in this sector. Fiji's mass tourism too has seen negative impacts, which are the results of a combination of numerous effects due to socio-economic, environmental and influencing factors such as political upheavals. Fiji's mass tourism's ever-increasing negative impacts make it imperative to analyze the ecotourism projects' status and influencing factors (especially political implications of Fiji's coups) to provide a future option of a sustainable form of tourism development.

This study comprehensively examines the revenue records of Fiji's Koroyanitu National Heritage Park (KNHP)—the Abaca ecotourist project—from 1993 to 2018 and reveals the connection between the ecotourist arrivals and the revenue generated vis-à-vis Fiji's political upheavals and their impact on mass tourism. The study makes observations about the impacts of Fiji's political coups on ecotourism by analyzing data and carries out a comparative analysis between Fiji's tourism data with KNHP's ecotourism records, using STATA and Augmented Dickey Fullar (ADF) test. It postulates a new scholarly argument of the success of ecotourism projects contrary to preceding literature which considers these projects as failures. While the paper primarily focuses on examining the impacts of Fiji's political upheavals with a comparative analysis between ecotourism and mass tourism data, it must be mentioned here that there are other factors of change too, for example, the global financial recession; the impact of regular environmental hazards; the change in government tourism policies; the racial difference between indigenous Fijians (*i-Taukei*) and Indo-Fijians; the land right problems and expiring of land leases; overdependence on overseas capital; the leakage of tourism profits; and donor-driven international influences. These are undoubtedly important, but they are not the focus of this paper. The main focus is the impact of Fiji's political upheavals on ecotourism, involving a comparative analysis of ecotourism and mass tourism data.

The demand for tourism is measured by the number of tourists and revenue generated through tourism; previous research has focused on the impact of Fiji's political unrest on mass tourism. But Fiji's political vulnerability in relation to ecotourism has not been explored for several reasons, including the lack of available primary data, difficulties in procuring it, the sensitive political atmosphere about research in rural areas and geographical inaccessibility of most ecotourism parks. Therefore, researchers are unaware whether Fiji's political unrest had similar or different impacts on ecotourism compared to mass tourism.

This research scrutinizes the first-hand records of Fiji's leading ecotourism destination, i.e., Abaca, along with national tourism data, in and after the period of Fiji's political instabilities, including records of tourist arrivals, invoices and revenue generated.

### 1.1 Study area

**Figure 1:**
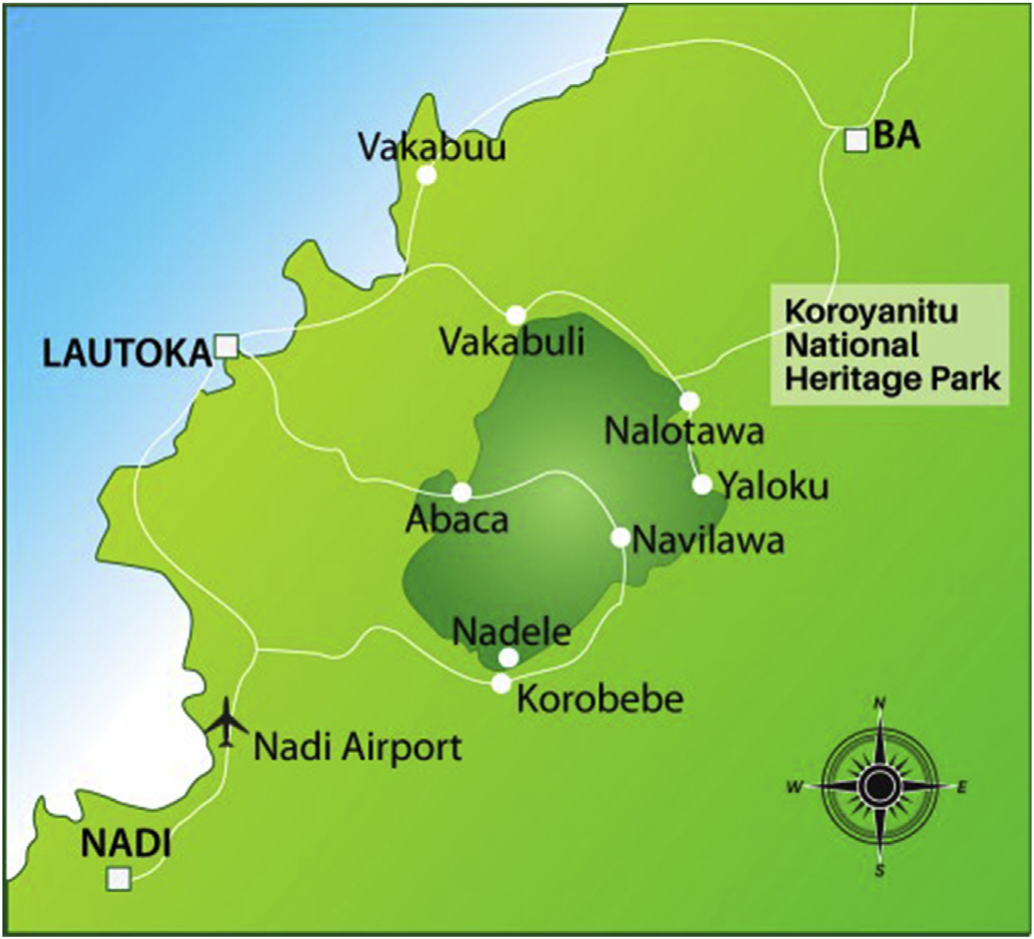
The Study area showing Geographical Location of Six Villages of KNHP, Western Viti Levu, Fiji. *Source:*Abaca: Ecotourism Guidebook, 1999. Published by Japan National Committee for Pacific Ecotourism Cooperation, Fiji.

The study area KNHP is in Lautoka, Western Viti Levu, which sprawls over an area of 250 km^2^ and includes tropical montane forest that has never been logged (Figure 1). Concerned about the impact of logging and mining interests, local chiefs and landowners set aside land to be protected and later to be incorporated in a heritage park in 1993. This park's land is owned by 18 landowners from six local villages, i.e., Abaca, Vakabuli, Nalotawa, Yaloku, Navilawa and Nadele/Korobebe (Zeppel 2006). The Abaca ecotourism project is supported by various stakeholders (Abaca 1999; Malani 2002), who have provided awareness and training to the villagers of Abaca in understanding sustainable land-use methods to preserve the environment. Many foreign donors helped in the foundation of this ecotourism park. This ecotourism park aims to provide supplementary income to the local communities and establish a better resource management regime. This research concentrates only on Abaca village, located north of Nadi, just 16 km south of Lautoka city (Abaca 1999).

Before narrating the tourism literature, an exemption of re-narrating the antecedent literature is made regarding describing diverse definitions of ecotourism, its discourse in Fiji, the benefits (environmental, socio-cultural and economic) and major international donors' details. However, the literature review primarily underlines the implications of Fiji's political crisis on mass tourism and superficially examines other influencing related factors. This shall provide an understanding to further compare the political implications factor with this ecotourism case study.

### 1.2 Literature review

#### 1.2.1 The impact of Fiji's political upheavals on mass tourism

Mass tourism in Fiji emerged in the 1960s. Gradually, by the mid-1980s, it witnessed the issues in foreign ownership, leakage in foreign capital, environmental degradation and negative ramifications of mass tourism, which encouraged the people to find alternate forms of sustainable tourism, i.e., ecotourism. However, this too suffered the concerns of a lack of coordination in ecotourism projects, a lack of conservation of the fragile environment, inadequacy in legislation and administration, limited skilled workforce, lack of proper tourism investment and insufficient infrastructural development. Fiji's four political putsches had negative ramifications on the country's tourism in the form of the embargo, tariff barriers, a cut in diplomatic relations (especially by neighbouring Pacific nations of Australia and New Zealand) and debarment from regional and international membership. The neighbouring nations imposed sanctions or gave warned its tourist to avoid travelling to Fiji, which went on to expose Fiji's overdependence on these nations for tourist inflow and revenue.

The literature has predominantly recognized that political instability has a direct implication on Fiji's tourist-oriented economy; it affects tourists' footfall, tarnishes Fiji's image as a tourist destination and forces the economy to adopt recovery policies (Sönmez 1998; Prasad, 2012). The research published in government records—for example, the Reserve Bank of Fiji (2004)—presents a picture of the superficial effect of coups and shows the capability of Fiji's economy to overcome them swiftly. It posits that natural disasters and political instabilities have a short-term impact on the EU and the UK markets; additionally, it postulates that ‘while the industry is vulnerable to internal [natural disasters and political instability] and external shocks [2009 Global Financial Crisis], it has been resilient and able to recover quickly from the adverse effects of various shocks’ (Wainiqolo and Shivanjali 2018).

In this vein, another paper perfunctorily observes that Fiji's political crisis is a major deterrent to the demand of tourism but attempts to emphasize categorically the constant average rate of growth in tourism (Katafono and Gounder 2004). Fiji's post-independence economy was sluggish and unstable, where political instability did not allow Fiji to achieve its full potential for economic growth (Mahadevan, 2019; Prasad and Tisdell 2006). Additionally, King and Berno (2006) indicate the long-term and short-term impacts of civil disturbances arising out of Fiji’ military coups (1987 and 2000) on tourism flow and tourism sector and also present the process of creating a tourism action group to bring together all stakeholders to overcome adversity and adopt various remedial crisis management strategies. Several other tourism crisis management strategies are highlighted by other researchers (King and Berno 2002). Globally, researchers have affirmed that politically motivated and violent events harm tourist arrivals (Neumayer 2004). These researches, however, are almost silent on the impact of Fiji's political upheavals on ecotourism.

It is imperative to comprehend the diverse contradictory arguments of the coups; impact on mass tourism and post-coup recovery pattern to make a subsequent comparison with ecotourism as undertaken in this research. Previous quantitative researchers' analysis (Table 5) state that after the 1987 coups, visitor arrivals fell by 26% and tourism earnings dropped by 21%; similarly, after the 2000 coup, arrivals fell by 28% and earnings by 29%, whereas Fiji noticed a marginal decline in arrivals after the 2006 coup as decreased by only 1% and through earnings fell by 5% due to discounts given by the tourism industry in the accommodation sector (Narsey 2004; Harrison and Pratt 2010; Vada-Pareti 2015). Fletcher and Morakabti's argument asserted that it took three years after the 1987 and 2000 coups for arrivals to recover (2008), while the 2006 one resulted in a two-year setback; further, they believe that the aftermath of the 2006 coup was milder due to shrewd marketing as visitors believed that the coup was non-violent and did not threaten them, so the losses were less (Harrison and Pratt 2010). Other scholars believed that this 1% decline of the 2006 post-coup effect in term of tourism activity might overall look small but it should be looked at in this context that ‘with Fiji experiencing strong growth (13.4%) year on year for the previous 5 years, thus, the decline in visitor's number be assumed to be close to 15% over that year’ (Fletcher and Morakabati 2008) (Table 5).

It is also argued that the 2000 political upheaval led to a substantial drop in tourism earnings, and the economy took one year to return to normal conditions (Harrison and Brandt, 2003; Prasad and Narayan, 2008). The 2006 December coup resulted in the plummeting of Fiji's gross tourism earnings. The interim government had paid considerable attention to the tourism sector since the military coup of 2006, as this industry only recovered since 2006. Albeit arrivals have increased but the gross tourism earnings have not increased proportionally (Prasad and Narayan, 2008; Prasad, 2012; Harrison and Prasad 2013). Therefore, after the 2006 coup, the Fiji government rightfully emphasized the importance of tourism and increased allocation of funds to Tourism Fiji, though the predictive forecast in 2011 may be difficult to achieve due to stiff competition from similar destinations (Harrison and Prasad 2013).

Conversely, other research indicated that the 1987 and 2000 coups had a short-term yet detrimental impact on the Fijian economy. The aftermath of the political instability resulted in Fiji's attempts to improve and regain its ‘safe’ image to attract potential investors back to the islands (Narayan and Kumar, 2005). Fiji managed to gain its reputation as the ideal tourist gateway (Pipike and Kia, 2012), though this may be a point of debate among researchers. Another research specified that the impact of political instability on tourism is far more severe than that of one-off terrorist attacks; sometimes, terrorist attacks increase tourism demand for those low to moderate political-risk countries, but tourism declines for high-level political risk countries, as seen in Fiji after the 2000 coup (Saha and Yap 2014). Political instability and the incidence of terrorism or coups lead to negative image building and leaves a legacy of unpredictable tourism demand. Moreover, the impact of terrorism can also lead to a negative image of the tourist destination, and it can only return to normalcy if the fear can be eradicated from the tourists' minds (Neumayer 2004). This compels tourism stakeholders to jointly take steps to revive tourism, as evident after the Fiji coups (Scott 1988). The border conflict and political instability between Eritrea and Ethiopia redirected the human force and revenue to the war exertion which disturbed the financial state of the neighbourhood individuals, subsequently bringing down the recurrence of the guests and prompting lower pay for protecting and building up the tourism destinations (Alam and Mishra, 2013).

Teye's (1998) Impact model, cited and enhanced by Rao (2002), explains the impact of Fiji's coups in detail (1987 and 2000) and the ramification of the instability on tourism in three broad areas. Firstly, the effectiveness of the Fiji Visitors Bureau (FVB) increased due to the financial support by post-coup Fijian governments to support tourism growth. FVB has been effective in marketing Fiji because of increasing visitor arrivals and foreign exchange earnings, contrary to Teye's argument of weakening of the National Tourism Organization in the post-coup period. Secondly, the flow of international tourist and the demand for tourist products declined that led to the formation of a crisis management team called Tourism Action Group (TAG) (and crisis action plans were made after coups) whose efforts were largely successful after the 2000 coup due to the joint efforts by the government who assisted with funds and the tourism industry that provided the expertise (cited by King and Berno 2006). Lastly, the development of tourism resources and attractions (supply of tourism products) and delivery of tourism services decline after coups (Rao 2002).

Concerning the perception of a tourist destination, the international media played a decisive role for Fiji when it broadcast images of political violence in Fiji in 2000, which led to numerous countries issuing an advisory to avoid travelling to Fiji. Many resorts became empty as tourists fled back home. It tarnished the image of the Pacific internationally in addition to issuing a range of international sanctions (Chand and Levantis 2000). Media and academics have questioned the freedom of media in Fiji during and after coups that worked under strict media censorship (Robie 2001; King and Berno 2006; Singh and Prasad 2008; Prasad, 2012). Fiji's tourist authorities have targeted travel agents and travel media to contain the negative impact of the military coup of 1987 (Scott 1988). During the 2000 coup, New Zealand's foreign minister said, ‘[T]he overthrows of Fiji's democratically elected government was intolerable, and Speight and his rebels can only be described as terrorists.’ The 2006 coup got the response of an international range of sanctions (Trnka 2008).

However, the United States terrorist attack of 9/11 made a positive impact on Fiji's dangling tourism image and gave it a new tag as a haven of tranquillity (King and Berno 2006). It is an irony that an attack on the US proved to be a blessing for other less politically unstable nations, such as Fiji. In this context, researchers state that the principle of someone's misfortune being another's gain should not be a reliable basis for planning (RBF Quarterly Review 2004; Neumayer 2004). Similarly, in the past, when Fiji suffered the two military coups of 1987, this violence benefited the Solomon Islands and North Queensland who advertised themselves as safe regional alternatives for tourism (Hall and O'Sullivan 1996).

While the feeling of fear and the perception of risk can destroy tourist arrivals, it has also been noticed that occasionally sites of terrorist atrocities or natural disasters can become attractions too, a phenomenon referred to as “dark tourism” (Korstanje and Clayton 2012). In response to the severe reduction of international tourists due to the 1987, 2000 and 2006 coups, TAG as an attempt to resurrect the image of Fiji as a “Pacific Paradise” in the face of this political turmoil (Trnka 2008) was commendable. However, the concept of dark tourism does not apply to small island states like Fiji.

Besides political uncertainties, Fiji's tourism has also been affected by other factors like land ownership issues and the rising concern of disputes over resort-based tourism (landowner versus the resort owner) and foreign-owned industry, which led to the leakage of tourism revenues.

This archipelago is prone to cyclones and natural disasters, especially during November–February, that see a reduction in the number of mass tourist visitors and revenue. The next hurdle for tourism was a severe decline of tourist arrivals in Fiji due to the 2009 global financial recession; however, this had little or no impact on other Pacific Islands due to the relative strength of the Australian economy (e.g., Cook Islands, Vanuatu, Solomon Islands and Papua New Guinea) (Harrison and Prasad 2013). The environmental crisis, political uncertainties, the declining state of other sectors of the economy, especially sugar, and global factors had forced Fiji to enforce their recovery plan of heavy discounting. Even though tourist arrival numbers were high, revenue derived from tourism had been low for some time, and many resorts were operating at a loss.

Besides Fiji's coups, other political events in Fiji have raised the eyebrows of its neighbours that led to a negative impact between the nations and their tourist inflow. For example, Australia has raised its voice on the abrogation of Fiji's 1997 constitution by President Ratu Josefa Iloilovatu Uluivadu on 10 April 2009, and also the re-appointment of Bainimarama as the interim prime minister. His policies were criticized by Australia as unacceptable, with severe economic implications that go beyond Fiji (Hayward-Jones 2009). Fiji was suspended from the regional Pacific Islands Forum and Commonwealth of Nations, which created uncertainty among tourists about Fiji's political stability. The 2009 global financial crisis aggravated this situation. In the wake of international pressure to return to democracy, this island finally re-established its parliamentary form of government through the introduction of the 2013 constitution and held the parliamentary elections in 2014. Since 2014, Fiji has seen political stability, which has led to growing mass tourism.

Without repeating the details of antecedent research data asserting the impact of Fiji's coups on the nation's conventional tourism in terms of its revenue and number of visitors, which show a general decline in tourism arrivals, especially during these political upheavals and the recovery period taken to reach the growth rate of pre-coup level (Katafono and Gounder 2004; Narsey 2004; Fletcher and Morakabati 2008; Harrison and Pratt 2010; Harrison and Prasad 2013; Wainiqolo and Shivanjali 2018), this research has concentrated on the 26 years of research data (1993–2018) of alternative tourism (ecotourism). The inception of this ecotourism park in 1993 makes it the year of commencing this research where the impact of Fiji's two 1987 coups cannot be compared. All applicable Fiji's political crisis are examined through quantitative ecotourism data to test the pattern after applying innovative methodologies.

## 2 Material and methods

This study is derived from the analysis of primary quantitative ecotourist visitors' data and secondary literature. The compiled data on different subjects have been tabulated. Synthesis, perusal and analysis of data have been carried out wherever it can explain the trend with the outcome of results and facts. Primary records include Abaca's tourist revenue records, including tourist invoices and the entries in the visitor books, further supported by personal interviews conducted with an ecotourism stakeholder, i.e., the manager of KNHP through *talanoa* (informal discussions in private/semi-private settings) sessions. The stakeholder will remain anonymous in accordance with the ethical guidelines of research confidentiality. Secondary information pertaining to the present study was based on published material on Abaca and subsequently supported by tourism policies of Fiji's Ministry of Tourism, recently published researches, magazines and other secondary materials. The descriptive method has been used for data analysis, and suitable parameters have been applied to make pattern assessments.

*Parameter One*: To distinguish between Fiji's mass tourists and Abaca visitors, the criterion is fixed as classifying Abaca visitors as ‘ecotourists’ that include mostly nature lovers and partially some mass tourists.

*Parameter Two*: The ‘ecotourists’ of Abaca consisted of both local and international visitors, but this study does not distinguish them. However, international visitors are the primary revenue source. They contribute the most to lodge fees, meal charges, in buying souvenirs, hiring guides and horses and also pay double the entry fees than a local visitor. A section of international mass tourists is also included in the ecotourist category, as these visitors also visit the ecotourism park during their regular mass tourism visit.

*Parameter Three*: To assess the pattern of ecotourist arrivals and earnings at Abaca during Fiji's coups, a standard is fixed to determine an ecotourism park's success/failure. Thus, favourable targets are selected for ecotourist arrivals at 900 and earnings at F$20,000 annually.

The obtained data sets have been represented by various statistical techniques, i.e., graphs and tables in an appropriate manner. To know the impact of visitors on revenue in Fiji and especially in Abaca village, the study tries to use simple regression analysis. Before applying regression, it is imperative to find stationary in variables. To draw a valid conclusion first, we need to confirm whether a series is stationary or not. This is done to avoid spurious regression.

There are various tests to check the stationarity in variables. Here, the ADF test is applied to check the stationarity. Simple linear regression is a model with a single regressor X that has a relationship with a response Y that is a straight line. This simple linear regression model can be expressed as:

(1) Y = β_0_ + β_1_X + ε

Where the intercept β_0_ and the slope β_1_ are unknown constants and ε is a random error term. Furthermore, MS Excel 2010 has been applied to examine the KNHP's ecotourist arrivals and revenue patterns. In the results, several observations are made after examining ecotourism quantitative data and comparing it with mass tourism researches.

## 3 Results

### 3.1 Tourism and ecotourism: A comparative analysis

It is believed that the negative consequences of mass tourism led to a search for innovative solutions that became one of the causes among many to move towards alternative tourism planning in Fiji. There is little evidence of successful ecotourism projects in the Pacific (Scheyvens and Purdie 1999; Farrelly, 2011). Forgoing research states that these ecotourism projects are not the solution for the lack of development and cannot be an alternative in the form of sustainable development; it is no quick fix for rural problems and, in fact, has its own problems (Harrison et al., 2003; Korth 2000). It cannot resolve the problems of large-scale tourism, and as Korth (2000) claimed, the representations of ecotourism have been politically motivated and ethnically biased towards the indigenous Fijian population.

***Observation 1:*** Based on research at the Abaca ecotourism centre, since 1993–1999, the number of visitors saw a gradual rise as it was emerging as a popular destination, but the third coup on 19 May 2000 tarnished Fiji's tourism image, impacting ecotourism. Under mass tourism, after May 2000, in the politically unstable state, Fiji witnessed a decrease of 30% of international tourist arrivals compared to 1999 (Harisson and Brandt 2003).

**Figure 2:**
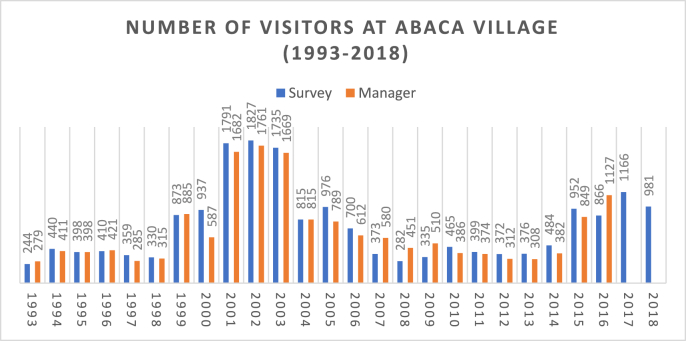
Number of visitors at Abaca Park (1993–2018). *Source*: Surveyed by the authors.

**Figure 3:**
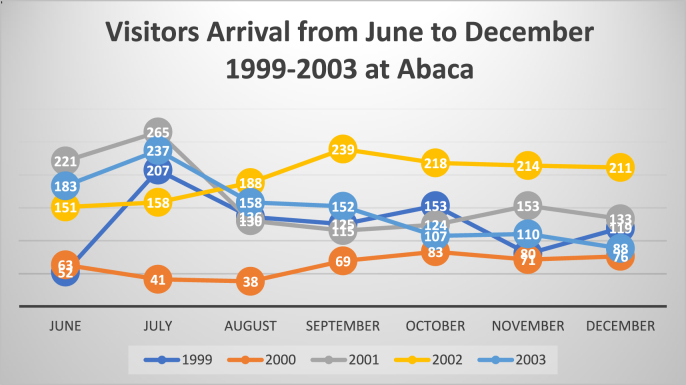
Shrinking Abaca visitors after the June–December 2000 Coup. *Source*: Surveyed by the authors.

This research observes the gradual rise of Abaca ecotourist arrivals from 1993 till the third coup (Figure 2); however, the sharp declining tourist number noticed in the months after the 2000 coup (Figure 3, despite the months of June till October which is the period of high tourist arrivals and revenue collection] and immediate recovery is noted in the next year, which is 2001. This ecotourism trend can be associated with the formation of a democratically elected government in Fiji that had positive implications on ecotourism. The primary (visitor registers and revenue invoices) and secondary data (provided by the manager of the park) are showing different statistics. In the *talanoa* session, Abaca's manager stated:

*The pathetic state of ecotourism park has deteriorated further due to the 2000 coup, and also after the exit of foreign aid. This tragic condition persisted after the 2006 coup.* (2 August 2017)

After the May 2000 coup, New Zealand Official Development Assistance (NZODA) programme reduced the funding of the KNHP project as it was reportedly disappointed with its progress, leading to improper management and a reduction in the tourist numbers (Harrison and Brandt 2003). However, the manager's words reflect an argument that is in contrast to propositions put forward by this research. This study presents a contradictory argument that within six months after the 2001 coup, this ecotourism park showed a tremendous rise in visitor numbers; additionally, the departure of foreign aid, especially after the 2000 coup, did not make a long-term impact on tourist inflow.

Traditionally, Fiji's tourism industry has witnessed the maximum number of visitors from Australia and New Zealand, but in 1987, the number of Australian visitors declined and made Fiji's source market highly diversified. Due to the political upheaval in Fiji, major source market countries like Australia, New Zealand, Japan and the USA advised people not to travel to Fiji or imposed travel bans. Fiji lacks a domestic market and depends mostly on international tourists (McVey and King, 1999). The ‘coup culture’ is a leading threat to the sustainability and growth of the tourism sector. Further, developed countries imposed various sanctions and advised their citizens not to travel to Fiji. This creates an atmosphere of unlawfulness, tension and insecurity that hampers the tourism industry of Fiji.

***Observation 2:*** When this ecotourism study is compared with the mass tourism data, it can be postulated that the period between 2001 and 2003 can be termed as a ‘golden period’ for ecotourism in Abaca as it saw a tremendous upsurge in visitor numbers (Figure 2). It has been stated that coups have short-term effects and after two or three years, the upward trajectory of Fiji's tourism continued (Harrison and Pratt 2010). However, this present study reflects a novel trend of the immediate rise of ecotourist arrivals in this park after six months of the 2000 coup.

**Figure 4:**
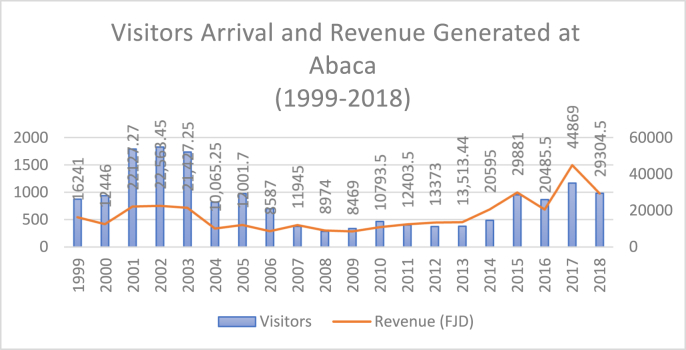
Number of Visitors, Revenue and Average Spending. *Source*: Authors' calculations. *Note*: *All monthly revenue invoices of some years were not available; so it is calculated on the average spending of last year multiplied by the number of visitors.*

***Observation 3***: In contrast, after Fiji's 2006 coup, the trend of the arrival of visitors at this ecotourism project declined tremendously as per Figure 4. There have been sanctions imposed by Australia and New Zealand on tourism, but this research states that these nations' ecotourists are the largest as they are the main market base for Abaca (based on Abaca tourist visiting register). So, the sanctions imposed by these nations also had an impact on Abaca ecotourism numbers. The 2006 coup generated negative international publicity that depicted the coup nation as volatile and several countries cautioned their citizens against travelling to Fiji. The political instability in Fiji after the 2006 coup carried on till 2014, which have implications in our research. This trend of decline was noticed in Abaca from 2007 to 2013 which was reflected in the number of arrivals and revenue generated with slight fluctuations (Figure 4). The international community looks up to the transition towards democracy—believed to a symbol of stability and tranquillity—in Fiji. Therefore, Abaca noticed a drastic tourist and revenue fall for eight to nine years after the 2006 coup.

On the contrary, despite these political upheavals, Fiji's mass tourism exhibited a steady growth of visitor arrivals between 1999 and 2009, with an annual growth rate in visitors at 4.2 % (Scheyvens and Russell 2012). A democratically elected government in Fiji reflects the image of political stability that is a prerequisite for capital inflow and investor confidence for sustainable development of tourism (Rao 2002).

***Observation 4***: This trend was noticed in Abaca's tourism where between 2001 and 2005, the ecotourist inflow escalated (Figure 2). Political turmoil, be it putsch or interim undemocratic government (2000 and 2006–2014), has adversely hampered the growth of Abaca ecotourism, whereas the period of elected governments in Fiji has seen relatively higher growth.

The available revenue invoices at the Abaca ecotourism project showed a massive upsurge in revenue collection in the year of Fiji's elected government in 2001, with a collection of approximately F$22,000 from approximately 1,790 visitors. The trend demonstrates a substantial increase in the number of visitors and revenue collection in the three years following the 2000 coup (Figure 4).

In the post-2006-coup period, the unstable political situation continued till the 2014 elections that had negative recompressions on Fiji's mass tourism as accepted by most researches (Table 1), but a paradoxical argument put forward states that the tourism industry uses the ‘negative publicity of coups in Fiji to claim more funds for marketing in order to deal with successive governments it seems remarkable coy’ (Harrsion and Pratt 2010).

**Table 1:** Number of visitors (ecotourists), revenue and average spending.

Year	Ecotourists at Abaca	Revenue (FJD) Abaca	Tourists in Fiji	Revenue (FJD) Fiji
1999	873	0.02 M	409,955	558.6 M
2000	937	0.01 M	294,070	387.2 M
2001	1791	0.02 M	348,014	447.9 M
2002	1827	0.02 M	397,859	554.9 M
2003	1735	0.02 M	430,800	638.6 M
2004	815	0.01 M	504,075	717.6 M
2005	976	0.01 M	545,145	733.2 M
2006	700	0.01 M	548,589	741.7 M
2007	373	0.01 M	539,881	784.1 M
2008	282	0.01 M	585,031	853.8 M
2009	335	0.01 M	542,186	848.9 M
2010	465	0.01 M	631,868	1194.4 M
2011	399	0.01 M	675,050	1286.5 M
2012	372	0.01 M	660,590	1300.0 M
2013	376	0.01 M	657,706	1318.2 M
2014	484	0.02 M	692,630	1512.2 M
2015	952	0.03 M	754,835	1683.4 M
2016	866	0.02 M	792,320	1823.2 M
2017	1166	0.04 M	842,884	1924.3 M
2018	981	0.03 M	870,309	2010.3 M

*Source*: Surveyed by the authors.

***Observation 5:*** The funds may have been used for mass tourism, but its benefits were not visible in the ecotourism project as the ecotourism inflow and revenue did not increase. The aforesaid researchers showed the post-2006 coup had immediate recovery in mass tourism, whereas Abaca ecotourist revenues recorded a consistent decline with marginal differences noted from 2006 till 2014.

Parameter number three (of securing the revenue of F$20,000) was achieved by the Abaca ecotourism park from 2014 onwards. Another observation is that the average expenditure per ecotourist after the fourth coup remained high since 2007 (above F$30; calculated by dividing revenue generated divided by tourists number); there was an exception in the year of 2009–2010 due to the global financial crisis, and in the year 2016 when Fiji was hit by the category 5 hurricane, Winston. Apart from the political instability, the global economic crisis and climate disasters too impacted the country's ecotourism market.

***Observation 6:*** Another pattern is posited from Abaca's arrivals and earnings (Figure 4) where the number of arrivals in comparison to revenue was mostly high after the 2000 coup in the democratic period. In contrast, after 2014, the revenue increased as compared to arrivals. Many factors impacted this trend, i.e., a rise in inflation, an increase in park entry fees, the growing popularity of this ecotourism site, higher demand for nature-oriented tourism, an increase in overnight stays and a rise in expenditure capacity of ecotourists.

The above descriptive analysis used time-series data covering the 1993–2018 periods, where the Mean for visitors at Abaca (VA) is 835.25 and Std. Dev. is 489.2733. Similarly, the mean for revenue at Abaca (RA) is .0165 and Std. Dev. is .0087509. In the case of visitors in Fiji (VF), the Mean is 586189.8 and Std. Dev. is 161598.4 whereas for Revenue in Fiji, the Mean is 586189.8 and Std. Dev. is 1056.95, respectively (Table 2).

**Table 2:** Descriptive analysis: Visitors of Abaca and Fiji.

Variables	Obs.	Mean	Std. Dev.	Skewness	Kurtosis
VA	20	835.25	489.2733	.8540618	2.739171
RA	20	.0165	.0087509	1.219559	3.702802
VF	20	586189.8	161598.4	.0125351	2.200688
RF	20	1065.95	513.9415	.4886791	1.934562

#### 3.1.1 Unit root test

To draw an effective conclusion, first, we need to check whether a series is stationary or not. This is done to avoid spurious regression. The Augmented Dicky Fuller test (Dicky and Fuller, 1981) is applied and the statistics indicate that both independent and dependent variables are stationary at first difference.

It reveals from the results that the p-value of the analysis is significant at less than 1%, which means the null hypothesis can be rejected. The coefficient is positive but very low, i.e., 0.000144. This, in turn, reflects that there is a significant and positive relationship between revenue and visitors in Abaca village. The analysis indicates that a 1 unit increase in visitors leads to a 0.000144 unit improvement in revenue. Further, the R-squared value is found as 0.4136, which specifies that 41% of the variation of revenue is explained by the model (Table 3).

**Table 3:** Regression result, Abaca.

Revenue	Coefficient	Std. Error	t-Statistics	Prob.
Visitors	.0000144	4.15e-06	3.46	0.003
Constant	.0004446	.00141	0.32	0.756


**R**^**2**^	0.4136	**Prob.**	0.0030

***Observation 7*:** The test statistics clarify that there exists a significant and positive impact of visitors on revenue in Fiji. P-value of the result is observed as less than 1%, which can reject the null hypothesis. The coefficient is positive, and the value stands at 0.001772. The work shows that if visitors are raised by 1 unit, then revenue will be increased by 0.001772 units. Furthermore, the value of R-squared is observed as 0.6691, which identifies that 67% of the variation of revenue is explained by the model. The correlation between visitors and revenue at Abaca (ecotourism) is 0.5017, which indicates a positive and moderate degree of association between visitors and revenue at Abaca. The correlation coefficient between visitors and revenue in Fiji (mass tourism) is 0.9709, which signifies a positive and strong degree of association between visitors and revenue in Fiji. A positive value of correlation (r) indicates a direct relation, a change in one variable is associated with a change in the other variable in the same direction. When the number of visitors rises, revenue also rises but in the case of Fiji, it is highly significant (Table 4).

**Table 4:** Regression result (Fiji).

Revenue (F)	Coefficient	Std. Error	t-Statistics	Prob.
Visitors	.001772	.0003022	5.86	0.000
Constant	33.4707	15.63569	2.14	0.047


**R**^**2**^	0.6691	**Prob.**	0.0000

### 3.2 Post-coup recovery assessment: resilient ecotourism

Rao (2002) argues that the ‘tourism industry is emerging as a symbol of the economic success of the Fijians and the Fijian nationalism associated with it.… [this industry is] benefitting the Fijians more than any other group, and therefore promoting and protecting the industry, particularly in times of crises [1987 and 2000 coups], fit well with Fijian nationalist sentiments’. The 2006 coup is believed to be between the political clash of interest of democratically elected Fijian Prime Minister Laisena Qarase and his party Soqosoqo Duavata ni Lewenivanua versus the Fijian military commodore, Frank Bainimarama. Fijian nationalist sentiment was not a major factor in the 2006 coup, as the threat of Indo-Fijians dominating has subsided.

***Observation 8:*** This argument related to mass tourism has shown similarity with Abaca ecotourism records. Abaca noticed a surge in visitors immediately after the 2000 coup (Figure 4), displaying a convincing association with Fiji's democratically elected government in 2001 and consolidating Fijian nationalist sentiments (Abaca ecotourism park is completely managed by indigenous Fijian). After the 2006 coup, the fear of Indo-Fijians superseding Fijians had subsided in national politics; thus, it took a long time to regain democracy. This presents a similarity with Abaca's recovery pattern that also became sluggish and took a long time to attain the pre-coup level in ecotourism after 2006.

After the declaration of elections in Fiji in 2014, the prospects of state-provided tourist security under a democratic regime helped Fiji regain international tourist confidence. Thus, from 2014 to 2018, the number of incoming ecotourists at Abaca village has shown a rising trend, accompanied by an equally high level of revenue generation. The revenue generated between 2014 and 2018 is almost above F$20,000 annually, while in 2017, the revenue peaked at over F$40,000.

***Observation 9:*** Table 5 demonstrates a comparative analysis of mass tourism (based on existing literature) and Abaca ecotourism data concerning Fiji's political upheavals and recovery period, stating rapid recovery in ecotourists inflow at Abaca just after 2000, whereas the post-2006 period noticed massive decline until democracy was revived through elections of 2014. During the 1987 coup, the Abaca ecotourism project assessment is not applicable, whereas after the 2000 coup, it took six months to revive ecotourism in terms of numbers and earnings (Table 5), which is dissimilar to the various claims made by preceding research for mass tourist data of recovery period that vary from one to three years in arrivals and two years in tourism earnings (Table 5). After the 2006 coup, the ecotourism project took nine years to recover and to reach 900 ecotourists, whereas after eight years, a revenue of F$20,000 was generated in contrast to various researches for mass tourist records that took one–two years for tourist arrivals and one year for tourist earnings to recover (Table 5). These mass tourism earnings are not considered proportionate to the tourist arrivals after the 2006 coup (Prasad and Narayan, 2008; Prasad, 2012; Harrison and Prasad 2013).

**Table 5:** Pre-coup level recovery analysis of mass tourism and Abaca ecotourism (in terms of tourist arrivals and earnings).

Years Coups	Based on Fiji’ Conventional Tourism Analysis (Antecedent Researches on Post-Coup Revival Duration)		Research Findings Based on Abaca's Ecotourism Project (Positive Target: 900 Ecotourist Arrivals; F$20,000 Earnings)	
	Tourist Arrivals Recovery	Tourist Earnings Recovery	Ecotourist Arrivals	Ecotourist Earnings FJD
1987	3 years (Narsey 2004; Harrison and Pratt 2010; Vada-Pareti, 2015) ∗1 year 6 months (King and Berno 2006) ∗Over 2 years (Rao 2002)	1 year (Harrison and Pratt 2010) ∗ Short-term and transitory effect on tourism expenditure (Narayan and Kumar, 2005)	Not applicable	Not applicable
2000	3 years (Narsey 2004; Harrison and Pratt 2010; Vada-Pareti 2015)	2 years (Harrison and Pratt 2010)	6 months	6 months
	∗ Over 3 years (Fletcher and Morakabati 2008; Rao 2002)			
	∗ 1 year (Harrison and Brandt 2003; King and Berno 2006; Prasad and Narayan, 2008)			
	% change from previous year	% change from previous year	% change from previous year	% change from previous year
	1999: 10%	1999: 16%	1999: 164.55	1999: NA
	2000: -28%	2000: -29%	2000: 7.33	2000: -12.81
	2001: 18%	2001: 17%	2001: 91.14	2001: 77.79
2006	2 years (Narsey 2004; Harrison and Pratt 2010; Vada-Pareti 2015)	1 year (Harrison and Pratt 2010) tourist arrivals increased but the gross tourism earnings did not increase proportionally with the increased numbers (Prasad and Narayan, 2008; Prasad, 2012; Harrison and Prasad 2013)	9 years (2006–2015)	8 years (2006–2014)
	∗1 year (Harrison and Prasad 2013; Prasad, 2012)			
	% change from previous year	% change from previous year	% change from previous year	% change from previous year
	2005: 9%	2005: 12%	2005: 19.75	2005: 19.23
	2006: -1%	2006: 1%	2006: -28.28	2006: -28.45
	2007: -1%	2007: -5%	2007: -46.71	2007: 39.11

*Source*: Authors' calculations.

Abaca's project manager, during a *talanoa* session, described the park's post-coup recovery pattern as following:

*The park has never recovered from political upheavals even after the 2006 coup, and it has been in a pathetic condition since then*. (Abaca Manager, 2 August 2017)

But this research based on quantitative analysis of primary data postulates a distinct post-coup recovery pattern for this ecotourism park.

***Observation 10:*** Furthermore, in terms of percentage change from previous years in the Abaca ecotourism park, the 2000 coup noticed positive arrivals of 7.33% due to a downfall in six months only after this coup and negative earnings of -12.81%, whereas a -28 % drop in tourist arrivals and a -29% drop in revenue collection in mass tourist statistics. Considering the 2006 coup's impact, the ecotourism project witnessed a -28% drop in both arrivals and earnings, whereas a minor change of a 1% rise in arrivals and a -1% fall in revenue the national tourism level. This reflects the Abaca ecotourism visitors and revenue records are opposite to national tourism data, as showing growth during the elected government periods after the 2000 coup and decline after the 2006 coup (Table 5).

The comparative analysis between Fiji's mass tourism and Abaca's ecotourism revenue collection shows that a democratically elected government has a significant role in enhancing the ecotourist arrivals and revenue collection in comparison to the trend observed in mass tourism. The ecotourism data assessment shows that the period of democratic governments between 2001–2006 and 2014–2018 witnessed a positive trend compared to the declining mass tourism trend after the 2000 coup and non-representative government from 2006 to 2014 (although any decline in mass tourism was temporary).

## 4 Discussion

This paper counters the arguments in the prior studies on the deteriorating condition of ecotourism in Fiji (Scheyvens and Purdie 1999; Farrelly, 2011; Korth 2000) and outlines the increase in revenue collection at Abaca during the rule of representative or democratically elected governments. The preceding scholarly arguments about Fiji's ecotourism park based on personal communication seems doubtful. As Korth stated, ‘Only [a] limited number of tourists participating in ecotourism activities, activities which are predominantly small in scale, the income generated has remained marginal within the tourist industry. For example, the income generated by two of Fiji's foremost landowner-operated ecotourism projects in Bouma and Abaca-barely reached F$12,000 annually by 1996 (Korth 2000). Abaca ecotourism has shown a positive impact in terms of tourist arrivals and revenue during the phase of Fiji's democratic government established after the 2000 and 2006 coups. Yet, Abaca remained marginal in Fiji's tourism, when compared with national mass tourism; however, it is significant from a micro-level aspect of ecotourism.

However, the number of ecotourists cannot match that of the conventional mass tourists, as the former are more research-oriented while travelling, i.e., they travel with a prior objective to explore the natural beauty rather than stay at high-to-medium-end resort-based accommodations. But the data of ecotourists and the revenue generated from ecotourism in Abaca can neither be generalized nor neglected, as it helps to build a ‘fresh insight’ in understanding the connection between alternative tourism and its revenue inflow with the regime of democratically elected governments in Fiji.

## 5 Conclusion

In the near future, ecotourism will not replace mass tourism or Fiji's resort-based tourism but shall provide an option for tailor-made trips for mass tourists and an avenue for ecotourists to satisfy their primary intent to travel. The research observations suggest ‘fresh insight’ in the study of ecotourism in Fiji by proposing that the principal reason for the decline in Fiji's ecotourism at Abaca tourist park is the political putsches and uncertain tenure during undemocratic non-elected governments that are associated with political uncertainty. Based on the above data analysis, it can be postulated that the periods from 2000 to 2006 and from 2014 to 2018 show potential for the development of ecotourism, manifested in a trend of a rising number of visitors and volume of revenue generated. The period of political instability, on the other hand, had a drastically adverse impact on Fiji's tourism, especially ecotourism and, in turn, delayed economic growth during the post-putsch recovery period.

The study has challenged all antecedent argument of the failure of ecotourism projects in Fiji, by analyzing primary data to demonstrate the success of this ecotourism park vis-à-vis Fiji's democratic status. Furthermore, after a comparative analysis of tourism with the ecotourism park data, the research reflects a distinct post-coup recovery pattern. The ecotourism income and arrivals trend shows a faster recovery in 2000 compared to the 2006 putsch, which is likely to have shown some possible similarities of a strong Fijian nationalism after 2000 in comparison to 2006 when the phobia of Indo-Fijians dominance was reduced.

STATA analysis and the ADF test make a correlation between visitors and revenue at Abaca (ecotourism) is 0.5017. The correlation between visitors and revenue in Fiji (mass tourism) is 0.9709. This demonstrates that both ecotourism and mass tourism are interdependent in relation to tourist arrivals and income generation. However, the ecotourism trend in terms of tourist arrivals and revenue generated shows a difference compared to mass tourism. MS Excel 2010 helped in making tables and figures based on tourist arrivals and revenue generated to present a new pattern of ecotourism that is affected by the existence of a democratic government in Fiji.

However, one must be mindful that the Abaca village ecotourism project's data analysis is by no means conclusive and comprehensive enough to be applied on a nationwide scale to Fiji's tourism data to derive generalizations. But the comparative studies cannot be overlooked as they speak volumes about ecotourism's condition during putsches. However, Fiji's political upheavals are the prime influencing factor for tourism, which included this ecotourism park as well; so all other secondary factors play a trivial role.

The political crisis in Fiji had threatened the country's image of ‘genuine friendliness’, ‘smiling faces’ and ‘paradise’ in much more when compared to other South Pacific destinations (Papua New Guinea and Vanuatu) that too have witnessed hostility and instability. Therefore, stability and peace associated with the democratically elected government are mandatory criteria for winning and retaining the ecotourists' trust and confidence in their safety. Nevertheless, the Abaca ecotourist park, together with other adjacent villages, can emerge as the biggest ecotourism destination in Fiji, provided the democratically elected governments continue in the country, which otherwise has been infamously tagged as ‘coup-coup land’.

## Declarations

### Author contribution statement

Sakul Kundra: Conceived and designed the experiments; Performed the experiments; Analyzed and interpreted the data; Wrote the paper.

Mumtaz Alam, Mohammad Afsar Alam: Performed the experiments; Analyzed and interpreted the data; Contributed reagents, materials, analysis tools or data.

### Funding statement

This research was funded by Fiji National University, Project Code URPC102.

### Data availability statement

Data included in article.

### Declaration of interests statement

The authors declare no conflict of interest.

### Additional information

Thanking Prof. David Harrison for giving expert suggestions and recommendations, and Prof. Tej Vir Singh for his valuable guidance for writing this article. Acknowledge the support of Mr. Joseph T. Lovulo for editing a Figure 1 map for this article.
